# The lower rate of bone and joint infection in patients with open extremity fractures associated with vaccination prior to injury: a propensity-matched cohort study

**DOI:** 10.3389/fphar.2025.1546191

**Published:** 2025-02-20

**Authors:** Wencheng Hu, Saiyu Shi, Junqing Lin, Tao Gao, Junjie Shen, Yi Sun, Haifeng Wei, Xianyou Zheng

**Affiliations:** Department of Orthopaedic Surgery, Shanghai Sixth People’s Hospital Affiliated to Shanghai Jiao Tong University School of Medicine, Shanghai, China

**Keywords:** bone and joint infection, vaccine, propensity-matched cohort study, trained immunity, fracture

## Abstract

**Background:**

Vaccines could strengthen the innate immune system in addition to conferring protection against their target pathogen via vaccine-induced immunomodulation, a phenomenon termed trained immunity. The purpose of the present study was to determine whether vaccination prior to injury is associated with a lower rate of bone and joint infections (BJIs) in patients with open extremity fractures.

**Methods:**

Patients with open extremity fractures treated at one hospital between January 2010 and December 2019 were identified. Incidental vaccine recipients and control cohorts were matched in a 1:1 ratio using propensity scores based on age, sex, anatomical location of the fracture, Gustilo–Anderson classification, body mass index (BMI), and diagnosis of diabetes. The primary endpoint was BJIs within 1 year after initial injury. Secondary outcomes were neutrophil counts and serum C-reactive protein (CRP) levels within 24 h of admission. Logistic or linear regression was performed to control for potential confounding factors when comparing primary and secondary outcomes.

**Results:**

Vaccine inoculation history was successfully collected from 6,338 patients, with only 83 patients receiving an incidental vaccine inoculation within 3 months before injury. After propensity score matching, demographic and clinical factors were well-balanced between cohorts (all standardized differences >0.1). After controlling for potential confounders, patients in the vaccine group were at a lower risk of BJIs after open extremity fractures (vaccine, 2/83 [2.4%]; control, 10/83 [12.0%), *p* = 0.011). Levels of circulating neutrophils and CRP were slightly increased in the vaccine group.

**Conclusion:**

Vaccine inoculation is associated with the lower BJI rate after open extremity fractures, and vaccinated patients might have a more robust immune response against bacterial challenges in terms of neutrophil and CRP levels after injury. Future prospective cohort studies and clinical trials are warranted to evaluate this finding definitively.

**Clinical Trail registration:**

http://www.chictr.org.cn/usercenter.aspx, identifier ChiCTR2000041093.

## Introduction

Bone and joint infections (BJIs) can lead to functional impairment and disability in patients and have a growing disease burden worldwide (Malizos, 2017). In the pathogenesis of BJIs, infections can be caused by direct wound contamination or by hematogenous microorganisms that colonize at the surface of the bone and implant to form a biofilm, leading to the persistence of BJIs (Stewart and Costerton, 2001). Patients with compromised immunity and related conditions, including aging, obesity, and diabetes, are at an increased risk of BJIs, following trauma and elective orthopedic procedures (Mears and Edwards, 2016; Gillespie, 1990).

Non-specific effects (NSEs) of vaccines go beyond the specific protective effect against the target disease. The mechanism of these effects is still unclear. It has been proposed that this effect is causally related to heterologous T-cell immunity and trained innate immunity (Welsh and Selin, 2002). For heterologous T-cell immunity, animal studies show that those infections, except for inducing pathogen-specific T-cell immunity, would also induce cross-reactive T cells with epitope sharing. Heterologous T-cell immunity would lead to improved clearance of the cross-reactive challenge (Selin et al., 2011). Except for heterologous T-cell immunity, researchers have paid attention to the trained innate immunity. The host immune system is classically divided into innate and adaptive immunity. The innate immunity is immediately activated after microbial challenge but responds nonspecifically to pathogens it encounters, while the adaptive immune system is slower to develop a response but is specific and forms immunological memory (Beutler, 2004). Thus, the innate immune system is more important in preventing the development of BJIs and its progression than the adaptive immune system (Lord et al., 2014).

Conventional wisdom was that immunological memory was an exclusive feature of the adaptive immune system. Recently, a novel phenomenon termed trained immunity was discovered, where the innate immune system can respond more effectively and broadly to infectious challenges after previous “training” with certain stimuli, including vaccines and immunostimulants such as lipopolysaccharide and β-glucans (Roden-Foreman et al., 2019; Netea et al., 2016). Vaccine inoculation against Bacillus Calmette–Guérin (BCG), smallpox, and measles appears to confer to patients nonspecific and enhanced immune protection against multiple infectious diseases for several months (Mina et al., 2015; Bartlett, 2003; Butkeviciute et al., 2018; Walk et al., 2019). Moreover, in preclinical studies, multiple pathogen-derived substances, including but not limited to heat-killed *Candida albicans*, zymosan, and lipopolysaccharide, have been used to train the innate immune system, enhancing the response against a second infectious challenge (Angulo et al., 2020; Quintin et al., 2012; Ciarlo et al., 2019). Zhu et al. explored the efficacy of immunity training in a murine model of periprosthetic infection and reported that after training with zymosan and lipopolysaccharide, with an increasing innate response, significantly fewer culture-positive joint samples were observed (Zhu et al., 2020).

To date, no clinical evidence in the field of musculoskeletal infection has been reported to support the phenomenon of trained immunity in humans. Thus, in the current study, we focused on determining whether incidental administration of a vaccine within 3 months prior to injury is associated with a lower infection rate in patients with open extremity fractures.

## Materials and methods

The procedures for collecting personal information from patients were approved and overseen by Shanghai Sixth People’s Hospital institutional ethics committee. The privacy information on participants was protected in accordance with local laws and regulations. Informed written consent was obtained from all patients, following the Declaration of Helsinki.

### Study design, setting, and participants

**FIGURE 1:**
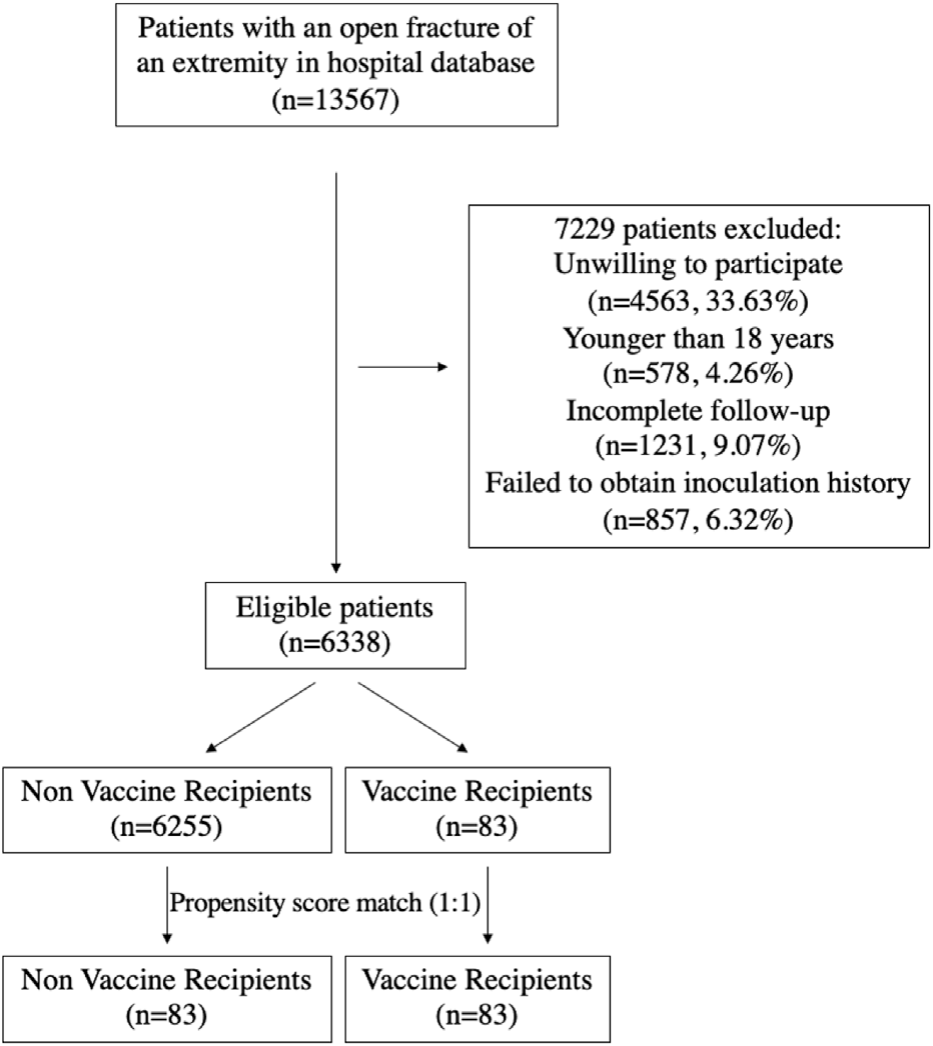
Flowchart of patients included and excluded from the study.

We conducted a retrospective study of all patients with open extremity fractures between January 2010 and December 2019. These patients were identified in the medical record database of Shanghai Sixth People’s Hospital affiliated with Shanghai Jiao Tong University School of Medicine. Because vaccine inoculation history was not recorded in detail in this database, research personnel contacted all eligible participants via telephone and/or instant messaging software to introduce and explain the purpose of the study. The personal inoculation history of eligible patients was commonly available at the local Centers for Disease Control and Prevention (CDC) online database. The research personnel informed eligible patients of the inquiry method and then collected and recorded their inoculation history. All the eligible patients were followed up for 1 year. Gustilo–Anderson classification (Brumback and Jones, 1994) was used to grade the open fractures into types I, II, IIIA, IIIB, and IIIC. We excluded patients if they were unwilling to participate (n = 4,563); younger than 18 years (n = 578); with incomplete follow-up, which was defined as less than 1 year from injury (n = 1,231); or if we failed to obtain inoculation history (n = 857). The inclusions and exclusions are listed in Figure 1.

### Surgical treatment

At the initial evaluation in the emergency department, all open wounds were irrigated with sterile normal saline and dressed with sterile gauze. The affected limb was temporarily immobilized for surgical preparation and further management. Patients then received a prophylactic intravenous injection of cephalosporin or clindamycin if allergic to penicillin. Additional antibiotics (e.g., aminoglycoside) were used when broader bacterial coverage was considered necessary at the discretion of treating surgeons. After thorough irrigation and debridement, the wounds were primarily closed or left open based on wound health and the degree of soft-tissue damage and contamination. When primary wound closure was not achieved, negative pressure wound therapy was used for temporary wound closure, and reconstruction (e.g., flap transfer and skin grafting) was performed in later stages to achieve wound closure. Fractures were either temporarily stabilized or received definitive stabilization. When definitive fixation was not achieved, the timing and type of definitive fixation were left to the treating surgeon’s discretion.

### Outcome measures

After propensity score matching (PSM), the medical records of the matched vaccine and control cohorts were reviewed by three authors (JL, JS, and YS). Consensus was achieved through discussion or, if necessary, by majority opinion. The primary outcome was BJIs within 1 year of injury. BJIs were defined according to the presence of one or more of the following: (1) apparent pus adjacent to the bone/implant during reoperation; (2) a draining sinus tract from the bone/implant; (3) isolation of pathogenic bacteria from a closed biopsy of bone/peri-implant tissue; (4) histopathologic diagnosis of bone/peri-implant tissue; (5) radiological findings suggesting BJIs; (6) receipt of additional antibiotics due to fever (>38°C), local pain, or tenderness. Blood cell counts and circulating C-reactive protein (CRP) levels were routinely examined at admission to our hospital, typically within 24 h before initial surgery. CRP and neutrophil were identified as effectors of infection and inflammation, and they play a role in innate immunity as an early defense system against infections (McDade et al., 2015; Musich et al., 2018). The CRP level was measured by scattering immunoturbidimetric assay, and the normal reference range was 0.00–10.00 mg/L.

### Statistical analysis

Continuous and categorical variables were presented as means ± standard deviations and counts (corresponding percentage), respectively, unless otherwise indicated. All statistical assessments were two-sided and were evaluated at the *p* < 0.05 level of significance. Baseline demographic and clinical variables were compared between the vaccine and control groups in the entire cohort. The Kolmogorov–Smirnov normality test was applied to analyze data normality. Fisher exact tests and Mann–Whitney U tests were used for categorical and continuous variables, respectively. PSM was used to adjust for differences in baseline characteristics between the vaccine and control groups at a 1:1 ratio by age, sex, the anatomical location of the fracture, Gustilo–Anderson classification, body mass index (BMI), and diagnosis of diabetes. These variables were selected based on their known association with the infection risk in orthopedic trauma. Age and sex were included as they influence immune response and trauma recovery, while older age and male sex were independent risk factors for infection (Mears and Edwards, 2016). BMI was accounted for due to its role in delayed wound healing and increased susceptibility to infection (Malizos, 2017). Anatomical location and Gustilo–Anderson classification were critical in capturing the severity of injury and soft-tissue damage, which are directly related to the risk of BJIs (Brumback and Jones, 1994). Lastly, diabetes was included as it significantly impairs immune function and predisposes individuals to infections. The standardized mean differences were calculated according to the procedure described in a previous report (Zhang et al., 2019). The nearest-neighbor matching method (Yao et al., 2017) was used, with a matching tolerance set at 0.01. Post-matching balance was evaluated by calculating standardized mean differences (SMDs) for all variables, with an SMD <0.1 indicating acceptable balance (Zhang et al., 2019). To reduce residual confounding, multivariable logistic regression was performed on the matched cohort to adjust for any remaining imbalances in covariates.

## Results

A total of 83 patients had a vaccine inoculation within 3 months prior to the injury. Among them, 46 patients were vaccinated against rabies, 20 against seasonal influenza, 6 against pneumococcus, 5 against hepatitis B virus, and 6 against other pathogens (4 against HPV virus and 2 against hepatitis A virus). A total of 37 patients were vaccinated the last dose within 1 month before surgery, 25 patients within 2 months, and 21 patients within 3 months. Before PSM, there were substantial baseline imbalances in covariates between the two groups (standardized difference >0.1) in age, sex, the anatomical location of the fracture, Gustilo–Anderson classification, BMI, and diagnosis of diabetes (Table 1).

**TABLE 1:** Univariate evaluation between vaccine and control groups of baseline characteristics before PSM^a^.

Variable	Control (n = 6,255)	Vaccine recipient^b^ (n = 83)	Standardized difference	*P*-value^c^
Age (yr) at injury, mean ± SD	41.8 ± 13.1	39.4 ± 13.4	0.183	0.095
BMI, mean ± SD	23.5 ± 3.5	22.8 ± 3.7	0.216	0.046
Sex, n (%)				
Male	4,697 (75.1)	54 (65.1)	0.220	0.036
Female	1,558 (24.9)	29 (34.9)		
Gustilo–Anderson type, n (%)				
I	453 (7.2)	8 (9.6)	0.245	0.257
II	1920 (30.7)	21 (25.3)		
IIIA	1770 (28.3)	19 (22.9)		
IIIB	1,266 (20.2)	24 (28.9)		
IIIC	846 (13.6)	11 (13.3)		
Length of operation (hours)	5.4 (3.2)	3.8 (3.7)	0.463	0.001
Length of hospital stay (days)	11.5 (6.3)	7.8 (5.0)	0.651	0.001
Anatomical site of the fracture, n (%)				
Lower limb	4,392 (70.2)	48 (57.8)	0.260	0.014
Upper limb	1863 (29.8)	35 (42.2)		
Domicile, n (%)				
Urban	4,178 (66.8)	58 (69.9)	0.067	0.639
Rural	2077 (33.2)	25 (30.1)		
Diabetes, n (%)	572 (9.1)	12 (14.5)	0.165	0.096
Hypertension, n (%)	907 (14.6)	13 (15.7)	0.031	0.128

^a^ PSM, propensity score matching; SD, standard deviation.

^b^ Patients received incidental vaccine inoculation within 3 months prior to injury and surgical repair.

^c^ Mann–Whitney U tests or Fisher’s exact tests were used, depending on whether the variable was continuous or categorical.

After PSM, the two cohorts were well-balanced in all baseline potential confounders (all standardized differences <0.1) (Table 2). After controlling for potential confounding variables, including age, the anatomical location of the fracture, Gustilo–Anderson classification, BMI, and diabetes diagnosis, the vaccine group had a reduced risk of BJIs compared to the control group [2/83 (2.4%) versus 10/83 (12.0%), *P* = 0.011; Table 3]. The most frequently isolated microorganisms from wounds were *Staphylococcus aureus* (control group versus vaccine group, five versus two). All the infection patients in the vaccine group were vaccinated for rabies (two of one vaccinated within 1 month prior to injury and the other within 2 months before injury). The patients in the vaccine group also had significantly higher concentrations of circulating CRP (51.7 ± 42.7 mg/L versus 39.5 ± 24.0 mg/L, *P* = 0.025) and neutrophils counts (6.8 × 10^9^/L ± 1.3 × 10^9^/L versus 6.0 × 10^9^/L ± 1.1 × 10^9^/L, *P* < 0.001) compared to the control group at admission. With our sample size, we identified no differences between the two groups in the interval from injury to wound closure (5.1 ± 4.0 days versus 5.4 ± 5.5 days, *P* = 0.722), interval from injury to definite fixation (6.4 ± 4.0 days versus 7.0 ± 5.4 days, *P* = 0.453), and the duration of antibiotic prophylaxis (9.4 ± 4.3 days versus 10.4 ± 5.2 days, *P* = 0.154).

**TABLE 2:** Characteristics of propensity-matched cohorts.

Variable	Control (n = 83)	Vaccine recipient^a^ (n = 83)	Standardized difference	*P*-value
Baseline values				
Age (yr) at baseline, mean ± SD	38.7 ± 13.9	39.4 ± 13.4	0.036	0.764
BMI, mean ± SD	23.0 ± 3.5	22.8 ± 3.7	0.056	0.600
Sex, n (%)				
Male	51 (61.4)	54 (65.1)	0.097	0.339
Female	32 (38.6)	29 (34.9)		
Gustilo–Anderson type, n (%)				
I	8 (9.6)	8 (9.6)	0.078	0.993
II	23 (27.7)	21 (25.3)		
IIIA	20 (24.1)	19 (22.9)		
IIIB	22 (26.6)	24 (28.9)		
IIIC	10 (12.0)	11 (13.3)		
Anatomical site of the fracture, n (%)				
Lower limb	54 (65.1)	48 (57.8)	0.099	0.339
Upper limb	29 (34.9)	35 (42.2)		
Length of operation (hours)	3.5 (3.2)	3.8 (3.7)	0.087	0.732
Length of hospital stay (days)	7.4 (5.0)	7.8 (5.0)	0.080	0.620
Domicile, n (%)				
Urban	56 (67.4)	58 (69.9)	0.039	0.867
Rural	27 (32.6)	25 (30.1)		
Diabetes, n (%)	10 (12.0)	12 (14.5)	0.074	0.647
Hypertension, n (%)	15 (18.1)	13 (15.7)	0.070	0.698

SD, standard deviation.

^a^ Patients received incidental vaccine inoculation within 3 months prior to injury and surgical repair.

**TABLE 3:** Comparisons of clinical outcomes.

Variable	Control (n = 83)	Vaccine recipient^a^ (n = 83)	*P*-value^c^
Infecting organisms			
*Staphylococcus aureus*, n (%)	5 (6.0)	2 (2.4)	-
*Coagulase-negative Staphylococcus*, n (%)	1 (1.2)	-	-
*Enterococcus faecalis*, n (%)	3 (3.6)	-	-
*Escherichia coli*, n (%)	1 (1.2)	-	-
Infection, n (%)	10 (12.0)	2 (2.4)	0.011^c^
Vaccination	-	All rabies vaccine	-
C-reactive protein (mg/L), mean ± SD	39.5 ± 24.0	51.7 ± 42.7	0.025^c^
Neutrophils (10^9^/L), mean ± SD	6.0 ± 1.1	6.8 ± 1.3	<0.001^c^
Days of wound closure, mean ± SD	5.4 ± 5.5	5.1 ± 4.0	0.722^c^
Days of definite fixation, mean ± SD	7.0 ± 5.4	6.4 ± 4.0	0.453^c^
Days of antibiotic prophylaxis^b^, mean ± SD	10.4 ± 5.2	9.4 ± 4.3	0.154^c^

SD, standard deviation; - no data.

^a^ Patients received incidental vaccine inoculation within 3 months prior to injury and surgical repair.

^b^ 24 h after soft-tissue coverage for all types of open fractures.

^c^ In each comparison, we used multivariable logistic or linear regression for categorical or continuous dependent variables, respectively, and controlled for potential confounding variables, including age, anatomical location of fracture, Gustilo–Anderson classification, BMI, and diabetes.

## Discussion

The innate immune system can be trained with vaccines and other immunity-stimulating substances to respond nonspecifically and more effectively to future infectious challenges (Roden-Foreman et al., 2019; Netea et al., 2016). For instance, one clinical trial demonstrated that BCG vaccination enhanced the immune response against *Plasmodium* parasites, which cause malaria in humans (Walk et al., 2019). Except for the BCG vaccination, another randomized study has shown that rabies vaccine provides a protective effect against unrelated respiratory, gastrointestinal, and febrile illnesses (Knobel et al., 2020). In addition, the influenza and pneumococcal vaccines were also proved to show a protective effect against other febrile illnesses (Kramskaya et al., 2019; Mann et al., 2014). The above studies suggest that vaccination would probably provide the protective effect against unrelated infection. In a preclinical study, researchers successfully conferred nonspecific protection against BJIs lasting up to 8 weeks in a mouse model via immune training (Zhu et al., 2020). Meanwhile, an animal study further found that transferring the trained platelets from immunity-trained mice would confer protection against BJIs (Gao et al., 2022). Based on the result of basic research in animals, we have reason to believe that trained innate immunity would provide the protection against BJIs. Nevertheless, no clinical evidence has been reported confirming the achievement of trained immunity to prevent BJIs. In the present study, we demonstrated that incidental vaccine inoculation with various vaccines could enhance immune responses to subsequent bacterial challenges and might reduce the risk of BJIs after open extremity fractures.

The current work had several limitations. First, this was a retrospective study in nature that inevitably has a selection bias. To mitigate this problem, we used PSM, followed by regression adjustment. However, unmeasured confounders, such as socioeconomic status; lifestyle factors; or other infections and diseases, such as malignant diseases, acute and chronic aseptic inflammation, and miscellaneous conditions that may increase CRP levels, could still have influenced the observed associations. Further discussion of these unaccounted factors is warranted to better understand their potential impact on infection risk and increase the validity of the data. Second, the efficacy of immune training could vary depending on the vaccine type. As shown in some observational reports, live vaccines [BCG, smallpox vaccine, measles vaccine, and oral polio vaccine (OPV)] would increase NSEs against vaccine-unrelated infections (such as sepsis and pneumonia), thereby reducing the overall mortality more than would be expected from preventing the vaccine-related infections (Benn et al., 2020). However, non-live vaccines (such as the DTP vaccine, hepatitis A/B virus vaccine, and HPV vaccine) seem to increase the possibility of vaccine-unrelated infections, which means that non-live vaccines would positively affect vaccine-targeted infections and may also have adverse effects on vaccine-unrelated infections (Benn et al., 2020). Further study in different settings is warranted to assess the contribution of varying vaccine types to trained immunity against unrelated disease, including BJIs. Third, only regular blood tests and acute reaction protein tests were collected in this research. There are many other indicators that would change after the innate immunity trained with vaccinate including IL-1β, TNFα, and IL6 (Kleinnijenhuis et al., 2012; Cirovic et al., 2020; Quintin et al., 2012). Thus, more comprehensive indicators tested before and after operation need to be collected in further perspective studies. Finally, the clinical application of trained immunity before open fractures seemed impractical. However, immunity training may have potential applications in elective procedures, such as arthroplasty and spine surgery. Although the prevalence of BJIs after arthroplasty and spine surgery is approximately 1% (Schuster et al., 2010; Gwam et al., 2017; Maradit et al., 2015), it is lower than that of open fractures, ranging from 7% to 22% (Patzakis and Wilkins, 1989; Bhandari et al., 2015; Costa et al., 2018). It is still meaningful and practical for some specific patient populations to get benefits from vaccine inoculation prior to surgery. Although potential challenges exist, how they could be addressed needs further consideration.

In preclinical studies, the injection of vaccine or immunity-stimulating substances (e.g., zymosan and lipopolysaccharide) has been widely used for studying immunity training (Netea et al., 2016). However, the safety of these immunity-stimulating agents remains questionable and untested. In contrast, approved vaccines have well-established safety profiles; thus, their translation to clinical use might be expected more rapidly for use as training agents. Nevertheless, weighing the benefits and risks, as well as the cost-effectiveness, of vaccination in patients scheduled for orthopedic surgery remains necessary. One possible strategy for avoiding this ethical dilemma in future trials is to choose vaccines as beneficial training agents, regardless of potential benefits related to BJI reduction. For instance, the seasonal influenza vaccine is widely recommended for healthy adults and immunocompromised individuals (Demicheli et al., 2018; Bosaeed and Kumar, 2018). Therefore, seasonal influenza vaccine might be a good candidate for future clinical trials investigating trained immunity for BJI reduction. Although more cohort studies need to be carried out to build the relationship between vaccine and BJIs, intervention studies are also needed. In addition, the feasibility and cost-effectiveness of incorporating vaccination into standard preoperative care protocols for high-risk orthopedic patients need to be explored.

Vaccination elicits trained immunity via the epigenetic modification of hematopoietic progenitors, leading to increased granulopoiesis in the bone marrow and an increased number of circulating neutrophils (Cirovic et al., 2020). A clinical study showed that the number of circulating neutrophils increased and remained at a high level for at least 3 months after BCG vaccination (Moorlag et al., 2020). In the present study, we corroborated this finding by observing an elevated circulating neutrophil count after innate immunity training with incidental vaccine inoculation, even after adjusting for confounding factors. In addition, after immunity training, neutrophils might respond to bacterial challenges more rapidly and robustly by producing more inflammatory cytokines (Ciarlo et al., 2019). Circulating CRP has long been recognized as a valuable marker of systemic inflammation because CRP is synthesized primarily in liver hepatocytes in response to the stimulation of most inflammatory factors (Sproston and Ashworth, 2018). Consistent with the classic theory of trained immunity, CRP levels were higher in our vaccine group than in our control group. Neutrophils, as phagocytes that ingest pathogens, were considered key responders to infection. In addition, CRP, as an inflammatory factor, could mediate the host immunity by interacting with Fcγ (Marnell et al., 2005). Based on these findings, the increased count of neutrophils and the high level of CRP after vaccination might provide infection protection for patients facing a high risk of BJIs. In addition, although a study has proved that the protection period from other unrelated diseases could last for up to 1 year after BCG injection (Aaby et al., 2011), the duration of protection in other types of vaccination against unrelated diseases remains under question. Thus, the protection duration conferred by different vaccinations against BJIs needs to be further considered in future studies, which would provide further evidence for future vaccine selection against BJIs.

## Conclusion

Vaccine inoculation, a typical method used for training innate immunity, was more likely to reduce the risk of BJIs after open extremity fractures. Vaccinated patients might have more robust immune responses against injury and bacterial challenge in terms of increased neutrophil counts and a higher concentration of CRP after injury. Future large sample-sized prospective cohort studies and clinical trials should be performed to further bolster this conclusion’s evidence. In addition, before suggesting a potential added indication for vaccination, further research is needed to examine the strength of the association between vaccine and infection risk.

## Data Availability

The original contributions presented in the study are included in the article/supplementary material; further inquiries can be directed to the corresponding author.

## Abbreviations

CRP, C-reactive protein; BJIs, bone and joint infections; BCG, Bacillus Calmette-Guérin; CDC, Centers for Disease Control and Prevention; PSM, propensity score matching.
